# Common Misconceptions Regarding Pediatric Auditory Processing Disorder

**DOI:** 10.3389/fneur.2017.00732

**Published:** 2018-01-23

**Authors:** Vasiliki Iliadou, Christiane Kiese-Himmel

**Affiliations:** ^1^Neuroscience, Medical School, Aristotle University of Thessaloniki, Thessaloniki, Greece; ^2^Phoniatric and Pediatric Audiological Psychology, University Medical Center Göttingen, Georg-August-University, Göttingen, Germany

**Keywords:** auditory processing disorder, children, hearing, central auditory processing disorder, hearing evaluation, auditory processing disorder management, hearing management

## Abstract

Pediatric hearing evaluation based on pure tone audiometry does not always reflect how a child hears in everyday life. This practice is inappropriate when evaluating the difficulties children experiencing auditory processing disorder (APD) in school or on the playground. Despite the marked increase in research on pediatric APD, there remains limited access to proper evaluation worldwide. This perspective article presents five common misconceptions of APD that contribute to inappropriate or limited management in children experiencing these deficits. The misconceptions discussed are (1) the disorder cannot be diagnosed due to the lack of a gold standard diagnostic test; (2) making generalizations based on profiles of children suspected of APD and not diagnosed with the disorder; (3) it is best to discard an APD diagnosis when another disorder is present; (4) arguing that the known link between auditory perception and higher cognition function precludes the validity of APD as a clinical entity; and (5) APD is not a clinical entity. These five misconceptions are described and rebutted using published data as well as critical thinking on current available knowledge on APD.

Hearing acuity may be difficult to assess in children and does not always reflect how a child “hears” in everyday life. The audiological test battery must be built around the pure tone audiogram and may include tympanometry, stapedial reflexes, auditory brainstem responses, and otoacoustic emissions. However, relying on such a test battery to measure auditory function in the setting of school or playground in children referred for auditory processing deficits is incomplete (1). Auditory processing test batteries should be employed in these cases to more fully evaluate hearing in an ecological manner and fully examine how well the child hears outside the ideal conditions of the audiology lab. Auditory processing evaluation is known to tap into the physiological function and integrity of the Central Auditory Nervous System (CANS) providing more comprehensive information about the integrity of the entire auditory system and the functional hearing status of a child.

Internationally, there is a marked increase in interest of pediatric auditory processing disorder (APD)—also known as Central Auditory Processing Disorder (CAPD)—with a fourfold rise in published research during the last decade (Scopus database). The increased interest has not yet translated into availability of clinical evaluation services. Specialized clinics providing diagnosis and management of APD are scarce in most countries. As an example, some European countries are still in the process of standardizing their auditory processing test battery or attempting to optimize inclusion of imaging in diagnostics [i.e., Ref. (2–4)]. Consequently, many children with undiagnosed APD are struggling both in school and during out of school activities, with negative impact on their phonologic and prosodic communication, academics, psychosocial behavior, and social skills.

There are at least five common misconceptions regarding APD that may be contributing to inappropriate or limited management in children experiencing APD. The intent of this paper is to present each misconception with a brief commentary on the underlying reasons for the misconception, and to provide, if available, published data that substantiates the authors’ perspective.

## Misconception 1: We Cannot Diagnose APD

This misconception reflects: (a) the lack of a universal consensus on how many and which auditory deficits constitute an APD and (b) the lack of a universal standard for test inclusion and specific cut-offs for attributing the APD diagnosis to an individual.

A gold standard for any given diagnosis is a widely used method considered to be the best available (5). Current clinical practice guidelines recognizing the inherent complexity of any given disorder rely on a battery of tests to diagnose a disorder (6). The best available up to date are those described in AAA 2010 guidelines (7). Keeping them as a basis one can still expand and refine by using new diagnostic techniques while still comparing them with the best available.

The Clinical Practice Guidelines of the American Academy of Audiology (7) state on page 22: “Several audiologists with many years of experience in clinical assessment of CAPD have independently agreed on a similar criterion for the diagnosis of CAPD; that is, a score two standard deviations or more below the mean for at least one ear on at least two different behavioral central auditory tests” [e.g., Ref. (8–10)]. This criterion, which was based largely on studies of sensitivity and specificity obtained using various cut-off values for various central auditory tests to identify known CANS dysfunction, has also been recommended by ASHA (11). The German Society of Phoniatrics and Pedaudiology (12) recommends that the APD diagnosis be applied when the individual scores are at least two SDs below the age norm of the reference population on at least two auditory measures accompanied by specific symptoms that cannot be explained by other factors, such as attention, cognition, or peripheral hearing impairment (13). However, the evaluation of the clinical significance of the results as well as the number of measures/tests that should be performed is left to the diagnostician. Research examining the minimum number of tests required to validate a test battery is needed. Recently, Musiek et al. (14) and Weihing et al. (15) examined this question and reported that a two test combination (frequency pattern test and low-pass filtered speech for children) was the option providing the best efficiency. A European consensus paper (16) identifies five criteria for making an APD diagnosis: normal hearing sensitivity (threshold ≤15 dB HL for each frequency between 250 and 8000 Hz in both ears), performance at or below 2 SD from the mean in at least 2 validated auditory processing tests that assess different processes in at least one ear (including non-speech sounds), presence of symptoms and risk factors related to APD, non-verbal intelligence coefficient >80 and ability of the individual to follow instructions in ideal conditions.

The authors identified several publications that seem to discard current APD protocols in school-aged children offering an abstract global approach that is not substantiated by appropriate research [e.g., Ref. (17)]. This approach may lead to failure to diagnose true hearing disorders (peripheral or central), and instead attribute symptoms to higher, supramodal, cognitive disorders. If proper audiological evaluation is not carried out, hearing loss can easily be misconstrued as an attention deficit (18). Moreover, some studies involve children with suspected but not diagnosed APD as participants (19–21). Children suspected of APD and children diagnosed with APD are two overlapping, but different groups. This means that conclusions reached about children suspected of APD cannot necessarily be generalized to children diagnosed with APD (22). Thus, there is a great need for additional research.

## Misconception 2: Valid Conclusions Concerning APD Can be Made Without Actually Testing Central Auditory Processing

This confusion stems from the argument that the current APD testing battery does not adequately control cognitive and especially language variables.

Listening difficulties may result from many deficits and disorders (hearing loss included) and some researchers use the term APD to describe “listening difficulties” in children (19). They assert that if we cannot accurately diagnose APD, then we can base our suspicion of APD on symptoms, or use auditory tests without documented efficiency (23). This leads to statements about suspected APD that are taken by some as equivalent to diagnosis of the disorder. But without examining an individual’s performance on an efficient central auditory test battery, it is impossible to sort out potential with cognitive measures nor reach conclusions regarding the true source of “listening difficulties” (16). It seems reasonable to assume that conclusions reached regarding APD based on diagnostic tests other than those that are of known efficiency and age-appropriate may not be contribute much to our understanding of APD (24).

## Misconception 3: If APD is a Secondary Diagnosis, then We Should Discard IT

This misconception is an inappropriate extrapolation from the appropriate need for differential diagnosis, and ignores complexity and co-morbidity of neurodevelopmental disorders.

This statement (24) may lead to children not fully managed regarding their auditory processing problems both in and out of the classroom. The notion that a single diagnosis is valid for each child is not realistic as any clinician evaluating children knows. For example, there are transient hearing impairments that may coexist with problems to sustain attention in the presence of distractions or other attentional control deficits with resulting deterioration of the symptoms exhibited. Of interest, neurodevelopmental disorders often cooccur with APD (25) producing deficits (*DSM-5*, p. 31, line 7) ranging from specific limitations in learning to global impairments of intelligence, social interaction, or quality of life (26). Due to the high likelihood of comorbidity, an audiologist specialized in APD evaluation and management should request reports from speech-language pathologists, special educators, teachers, and psychologists—depending on a child’s difficulties—in order to more effectively elect central auditory processing tests to administer and plan for management and treatment [e.g., Ref. (27, 28)]. There are practical guidelines (29) that the clinician can use to minimize confounding cognitive and language variables for an adequate differential diagnosis of APD.

## Misconception 4: APD Reflects Cognitive Deficits

This statement is similar to the nature or nurture debate.

Auditory perception is a known contributing factor to the assessment of cognition (30), especially since most clinically used tests for verbal cognition rely on an individual’s verbal reproduction of an item presented auditorily. Adults with peripheral hearing loss have been misdiagnosed as cognitively impaired due to this often overlooked contributing factor (31, 32). Short-term memory (STM) assessment is one of the most obvious measures that may lead to incorrect conclusions in the presence of uncorrected auditory dysfunction. In the absence of adequate hearing/audibility of test items, an individual may be incorrectly classified as having lower verbal STM. When hearing acuity is corrected (e.g., using a hearing aid, raising the intensity of the stimuli, or improved speech to noise ratio), the assessment will provide a more accurate measure of STM. Similarly, performance deficits on cognitive measures such as STM would not be an adequate explanation for the source of disparate disorders, such as language impairments, dyslexia, dyscalculia, ADHD, learning problems, or cognitive difficulties seen in autism. Moreover, correlation between APD and cognition does not impute a specific causal direction (33). It is essential that in the papers where APD is diagnosed and not just suspected, IQ cannot explain auditory processing deficits (15, 34). In a recent paper, the same applies for patients referred for APD testing (35).

## Misconception 5: APD is Not a Distinct Clinical Entity

This assertion relies on all previously refuted misconceptions 1–4.

The accumulating body of research on APD over the last two decades led to the classification of APD in International Classification Disorder System (ICD), both the 10th and 11th editions (36, 37), as an “ear disease,” thereby confirming that it is a physiological entity requiring medical attention. Moreover, APD is an accepted clinically recognized entity by many audiological societies throughout the world (7, 11, 12, 38–43). It is of interest that those researchers arguing that APD does not exist tacitly accept the existence of APD symptoms (19, 24, 44). They attribute these symptoms to attention and cognition without, apparently, recognizing the auditory perceptual contributions to cognition.

## Conclusion and Future Research

The authors recognize that differential diagnosis is made difficult due to the overlapping symptoms across neurodevelopmental disorders and APD as well as many clinicians’ limited education in interpreting results of auditory processing tests and disentangling them from results obtained by the multidisciplinary professional team, which sometimes are not even available for review. In future, these diagnostic challenges should be addressed through continuing in-depth education of audiologists and other health care professionals who are responsible for evaluating and managing children with APD. Audiologists would benefit from additional research with children diagnosed with APD focused on comparing new evaluation techniques with clinically validated approaches to ensure that new approaches meet the essential psychometric requirements and documented sensitivity to CANS lesions. The potential interactions between these new tools and cognitive, attention, and language indexes must also be examined. Standardizing these novel techniques across typically developing children and children with known brain pathology will provide further validation needed for clinical adoption to more effectively diagnose and treat APD.

## Author Contributions

VI had the original idea of the paper and have written the first draft together with CKH. She subsequently edited and enhanced it to produce the final submitted form. CKH added arguments and enhanced the draft to produce the final submitted form.

## Conflict of Interest Statement

The authors declare that the research was conducted in the absence of any commercial or financial relationships that could be construed as a potential conflict of interest. The reviewer JW and handling Editor declared their shared affiliation.
